# Understanding the scalability of personalised monitoring within indoor spaces

**DOI:** 10.1038/s41746-023-00854-3

**Published:** 2023-05-31

**Authors:** Graham Coulby, Alan Godfrey

**Affiliations:** Department of Computer and Information Sciences, Faculty of Engineering and Environment, Northumbria University, Newcastle Upon Tyne, NE1 8ST UK

**Keywords:** Translational research

## Abstract

Health and wellness/well-being are multifaceted topics further complicated when trying to understand the environmental impact. Typically, there has been a one size fits all approach when trying to understand the 3-way interaction, but that is a limited approach. Equally, measurement (of each) has often used a limited set of outcomes during short periods to provide insight. A more robust understanding of health and well-being within environments may require longitudinal/continuous assessment that holistically targets individuals. Therefore, there is a growing requirement for careful data management, individual-first methodologies, scalable research designs and new analytical approaches, e.g., artificial intelligence. That presents many challenges but interesting research opportunities for the field of digital medicine.

## Introduction: data, data everywhere

Global urbanisation means people are spending much of their time within indoor environments and in high proximity, which may negatively impact health and wellness/well-being^1^. Accordingly, workplace health and well-being are important societal, industrial, and personal considerations^2^. The multifactorial construct of each topic (health, well-being and environment) and numerous arising outcomes makes this a challenging area of research to find causal relationships. Traditionally, measurement tools for each have been limited. For example, well-being is often assessed via pen-and-paper questionnaires at the end of a study, while environmental monitoring uses single-point location devices to continuously survey the indoor environmental quality across a large space. There is a need within the field of digital medicine to measure outcomes robustly and holistically for the individual.

Recently, Srinivasan et al. conducted a multi-modal field study to monitor associative patterns between sound levels and physiological well-being across a workplace population, examining 231 participants over three days^3^. That study investigated the effect sound levels have on physiological well-being by creating a platform to triangulate data from environmental monitoring devices, wearable heart rate and activity monitors, as well as physiological perception surveys. The authors found that optimum physiological well-being was observed when sound pressure levels were ≤50 dBA. Srinivasan’s study highlights two key implications that are important to discuss: (1) challenges surrounding the scalability of digital medicine and (2) the heterogeneity of individuals.

## Scalability: data accumulation

Despite the relatively short measurement period (3 days) adopted in the study, >200,000 accumulated minutes of wearable data were captured, which were aggregated with over 30,000 measurements from sound and survey data. This highlights challenges around the scalability of personalised environmental monitoring, as a wealth of data were generated in a short time from only one environmental and two physiological outcomes. If three digital sources alone can generate data at that scale, then longitudinal, holistic measurements of outcomes can quickly become an almost exponential challenge. Yet, as this research transitions into patient care pathways, there becomes a need for longitudinal assessments that monitor environmental changes over time to understand the effect those changes have on individuals^1^.

### Heterogeneity

This had a significant impact on Srinivasan’s study, as authors were required to apply separate models to examine the heterogeneity of outcomes across the population^3^. They used partial pooling with Hierarchical Bayesian models to allow for variation in parameters at an individual level while estimating parameters across the population. Consequently, while digital medicine is already an extremely multifaceted area of research, heterogeneity adds significant complexity to the equation. Many aspects of health and well-being can be inherently unquantifiable (e.g., happiness, experience and privacy), and this is, *in part*, due to the subjective and personal experiences surrounding the outcomes under observation^4^. Perhaps there is scope to approach this problem with another method, where the heterogeneity of individuals is elevated to the core within this research domain, to take an individual-first approach. That creates a need to explore methodological approaches which may be better suited to individualised measurements.

### Individual-first research

Single-case designs (SCDs), *also known as n-of-1 study designs*, could play a useful role, as they focus on the systematic and repeated data collection from individuals over time^5^. SCDs take an individual-first approach to research but can be pooled to gain a wider understanding of a population. The longitudinal nature and individual-first approaches *adopted in SCD research* can expose causal associations that occur between outcomes over time^6^. This could be used to elevate heterogeneity as a core research focus. Consequently, researchers could potentially explore the unquantifiable aspects of digital medicine without worrying about subjective biases across a population.

While SCDs could be used to develop personalised measurement approaches that elevate heterogeneity in environmentally focussed health and well-being studies, the method could be oppositional towards scalability. Specifically, individualised measurements require more sensors, which in turn creates more data for analysis^7^. Increasing the number of environmental sensors has significant cost implications. While fashion-technology trends (such as smart watches, rings and clothes) are driving down costs of multi-modal wearable technologies^8^, environmental sensors are often expensive and have limited sensing modalities^1^. Accordingly, multiple dedicated sensors may be required to holistically observe an environment. Alternatively, low-cost sensors have the potential to address scalability concerns by providing cheaper technological solutions, which can be validated as fit-for-purose^9^. However, as data management scales, the underlying analytical methods must also scale to support the data influx.

To longitudinally observe holistic, heterogeneous outcomes at an individual level, the scale of multi-variate analyses must be carefully considered. As the quantity of data scales, it is important to adjust the analytical focus to explore both micro/quality and macro/quantity trends within the data^7^. In much the same way Srinivasan’s study applied two models to account for individuality versus heterogeneity of a population^3^, different analytical approaches are required for longitudinal and intra-day evaluations. With current approaches, macro-level assessments can be useful for identifying points of interest in longitudinal datasets to reduce the burden of analysing longitudinal, high-frequency, intra-day data^7^. However, as affordable technologies permeate research in this area, outcomes can scale, and longitudinally can increase to potentially support continuous assessment at an individual level. Therefore, it is likely that treatment processes, which utilise individualised and environmentally focussed digital medicine, will scale to a degree whereby artificial intelligence becomes a requirement, a tool to automate data analysis.

## Concluding thoughts

Overall, digital medicine will continue to be a complex, multifaceted, and subjective area of research. By adopting mature individual-first methodologies, longitudinally can be explored within treatment processes to potentially expose richer insights into the nuanced interplay between health, well-being and the environment. That may enable research and pragmatic patient care that can scale to include a more holistic set of outcomes. However, this will require careful data management strategies to manage how data will scale as wider ranges of outcomes and longitudinally/continuity are included. Furthermore, maintaining awareness of how outcomes adapt over time adds additional complexity to an already complex area of study. This presents an exciting set of challenges that can drive research opportunities and health interventions for shaping the future of digital medicine.
